# Cleft Lip and Palate in Four Full-Sib Puppies from a Single Litter of Staffordshire Bull Terrier Dogs: An Anatomical and Genetic Study

**DOI:** 10.3390/ani13172749

**Published:** 2023-08-29

**Authors:** Jakub J. Ruszkowski, Joanna Nowacka-Woszuk, Tomasz Nowak, Jedrzej Rozynek, Katarzyna Serwanska-Leja, Maciej Gogulski, Pawel Kolodziejski, Marek Switonski, Maciej Zdun, Izabela Szczerbal

**Affiliations:** 1 Department of Animal Anatomy, Poznan University of Life Sciences, Wojska Polskiego 71C, 60-625 Poznan, Poland; jakub.ruszkowski@up.poznan.pl (J.J.R.); katarzyna.leja@up.poznan.pl (K.S.-L.); 2 Department of Genetics and Animal Breeding, Poznan University of Life Sciences, Wolynska 33, 60-637 Poznan, Poland; joanna.nowacka-woszuk@up.poznan.pl (J.N.-W.); tomasz.nowak@up.poznan.pl (T.N.); jedrzej.rozynek@up.poznan.pl (J.R.); marek.switonski@up.poznan.pl (M.S.); 3 Department of Preclinical Sciences and Infectious Diseases, Poznan University of Life Sciences, Wolynska 35, 60-637 Poznan, Poland; maciej.gogulski@up.poznan.pl; 4 University Centre for Veterinary Medicine, Poznan University of Life Sciences, Szydłowska 43, 60-637 Poznan, Poland; 5 Department of Animal Physiology, Biochemistry and Biostructure, Poznan University of Life Sciences, Wolynska 35, 60-637 Poznan, Poland; pawel.kolodziejski@up.poznan.pl

**Keywords:** *ADAMTS20*, cleft lip, congenital malformation, *DLX6*, dog, genes, *MYH3*, palatal clefts

## Abstract

**Simple Summary:**

Orofacial clefts occur due to incomplete fusion of the tissues forming the lip and palate during embryonic development. There are three categories of such cleft: cleft lip (CL), cleft palate (CP), and cleft lip and palate (CLP). Orofacial clefts are among the most common congenital malformations found in dogs. Puppies with this condition suffer aspiration pneumonia, malnutrition, failure to thrive, and death if not treated. Surgical correction of canine CLP is increasingly common. Detailed radiological anatomical studies of the skulls of affected puppies of different breeds are thus required to plan medical treatment. Moreover, genetic studies are needed to search for the genetic background of this developmental anomaly in dogs since it is well-known that orofacial clefts are caused by genetic and environmental factors. There are numerous candidate gene mutations that cause these abnormalities in mammals. These include *ADAMTS20*, *DLX6*, and *MYH3*, which have been examined in the present study, though no DNA variants are detected. In conclusion, the molecular background of this developmental abnormality, observed in four out of seven puppies of a single litter, remains unknown.

**Abstract:**

Cleft lip and palate (CLP) is a well-known congenital defect in dogs, characterized by abnormal communication between the oral and nasal cavities. Its incidence rate is high and affects all dog breeds. The etiology of CLP is thought to be multifactorial, caused by both genetic and environmental factors. In this study, four puppies out of seven from a single litter of Staffordshire Bull Terrier dogs with craniofacial abnormalities were anatomically and genetically examined. Classical anatomical preparation, dyed-latex-injection of the arterial vessels, and cone-beam computed tomography were used. The puppies showed variations in their observable abnormalities: three of them had a complete cleft of the palate on both sides, while one puppy had a cleft on the right side only. Cytogenetic analysis showed a normal diploid chromosome number (2n = 78,XX or 78,XY) in the studied animals. Known genomic variants of CLP were examined in the *ADAMTS20*, *DLX6*, and *MYH3* genes, but no mutations were identified. Further studies are needed to identify the breed-specific genetic variants associated with canine CLP.

## 1. Introduction

Orofacial clefts are defined as congenital anomalies of the lip, the palate, or both, which occur due to the incomplete fusion of tissues during embryonic development [1]. Cleft lip (CL), cleft palate (CP), and cleft lip and palate (CLP) are the most common types of orofacial cleft. In humans, orofacial clefts may occur alone or as a feature of various syndromes. The incidence of nonsyndromic or isolated CL with or without CP is 1 per 700 live births in humans [2]. These complex birth defects result from genetic variation, environmental exposure, and other health interactions. A CL, with or without a CP, is the most common craniofacial birth defect in dogs [3]. It has also been described in other domestic animals, such as cats [4], horses [5], and cattle [6,7]. It has been found that CP is more common in purebred dogs than in mixed breeds [8]. All canine breeds can be affected, though brachycephalic dogs (e.g., English and French Bulldogs, Boston Terriers, and Shih Tzu) are especially predisposed [9]. In retrospective studies, CP has been recognized as the most common malformation, with a prevalence of 1.3% [9]. Its frequency varies by breed, with 0.11% of Pyrenees Shepherds, 0.6% of Boxers, and 2.2% of Beagles affected [10]. It has also been shown that breeds from the mastiff/terrier genetic cluster are more predisposed to orofacial clefts [1].

In healthy dogs, the palate (*palatum*) is a bony-membranous structure separating the nasal cavities and the nasal part of the nasopharynx from the oral cavity and the oropharynx. It comprises the hard palate (*palatum durum*) and the soft palate (*palatum mole*). The hard palate is a nearly flat bony structure formed of paired processes of the incisive, maxillary, and palatine bones. The soft palate is a membranous structure that continues caudally from the hard palate. A cleft is an abnormal fissure in a body structure resulting from the failure of parts to fuse during embryonic development. Palatal clefts may spontaneously develop during pregnancy or may be induced by teratogens or surgery [11]. The embryological mechanisms are not completely understood, but it is hypothesized that a lack of apoptosis or cellular transformation could be involved in the background of clefts.

Since some dog breeds are more prone to cleft defects than others, the genetic component of this condition needs to be taken into account. Moreover, recurrent cases of this condition in a pedigree strongly indicate a genetic condition. In human studies, many gene variants have been identified as associated with nonsyndromic CP (summarized by [12]). These genes are crucial to a range of processes such as cell–cell adhesion (e.g., *IRF6*, *FOXE1*), cell proliferation (e.g., *MSX1*, *GRHL3*, *PAX7*, *TBX22*), cell migration, and processes involved in folate and homocysteine metabolism. Genes responsible for syndromic forms have also been considered candidates for nonsyndromic CP [12]. Extensive genome-wide association studies have facilitated the identification of several dozen common risk loci [12]. However, it is estimated that ~70% of the estimated heritability for nonsyndromic CP remains unexplained [13]. Whole-genome sequencing thus provides an opportunity for detecting novel gene mutations [14]. In dogs, mutations in two genes, *DLX6* and *ADAMTS20*, in Nova Scotia duck tolling retrievers (NSDTR) presenting a variety of cleft defects have been described [3,15]. Two fully linked mutations in the *MYH3* gene that causes recessive CP in French Limousine cattle have also recently been reported [6].

The present report describes an extensive anatomical and genetic evaluation of four puppies originating from a single litter of Staffordshire Bull Terriers with abnormal craniofacial development. Fragments of three candidate genes for CLP (*ADAMTS20*, *DLX6*, and *MYH3*) were sequenced to seek DNA variants associated with the abnormality.

## 2. Materials and Methods

### 2.1. Animals

**Figure 1:**
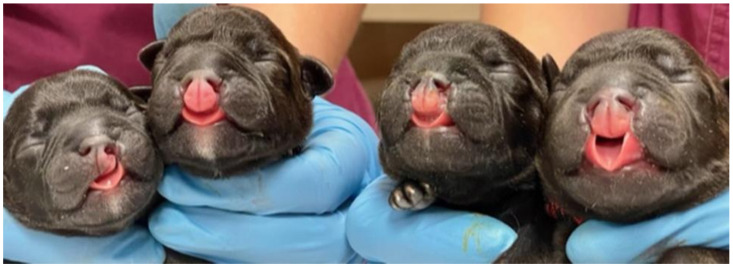
Orofacial clefts in newborn puppies. The figure shows cases 3, 1, 2, and 4, respectively.

The study was conducted on four Staffordshire Bull Terrier dogs from a single litter (n = 7). The mother, a three-year-old female dog, was artificially inseminated (AI) by a veterinarian specialized in theriogenology. Before AI, progesterone levels were measured, and ultrasound examination of the ovaries was performed regularly. Two AIs were carried out with a 24-h interval. It was the first pregnancy and showed normal progression. Ultrasound fetometry was performed in the fourth and sixth weeks of pregnancy; no abnormalities were observed. The puppies’ heart rates (HRs) were measured and were above 230 bpm. The pups were delivered at 63 days after AI by Cesarean section. There were seven puppies, including five males and two females. Three males and one female had orofacial clefts (Figure 1). No other developmental abnormalities were observed. The other three siblings of the litter did not show any congenital malformations. The affected animals were euthanized at the owner’s decision. The dogs were premedicated with intramuscular injection of dexmedetomidine (Dexdomitor, 0.05 mg/kg) and ketamine (Ketamidor, 10 mg/kg) followed by intravenous injection of pentobarbital (Exagon, 100 mg/kg).

### 2.2. Anatomical Evaluation

Three anatomical techniques were used in this study, including classical anatomical preparation, dyed-latex-injection of the arterial vessels, and cone-beam computed tomography. Cone-beam computed tomography scans (Fidex Animage, Pleasanton, CA, USA) of all four dogs were performed at the University Centre for Veterinary Medicine in Poznan, Poland. After the examination, the scans were reconstructed to obtain a 3D model of the skull using 3D Slicer software (version 5.0.3.).

Two randomly selected dogs with CLP (Case 1 and Case 4) were used to obtain angiological specimens. After cutting the abdominal and thoracic cavities open, the aortas were injected with red-dyed liquid latex LBS3060. After the injection, the preparations were cured in 5% formaldehyde solution for ten days. Before preparation, the specimens were flushed with tap water for 48 h to remove excess formaldehyde. The preparation was conducted using a surgical scalpel and anatomical forceps. The mucosa and muscles of the oral cavity were then cautiously cut to visualize the stained arterial vessels. After cleaning excess connective tissue from the specimens, the arterial pattern of the palate region was described.

The names of the anatomical structures used in the description were standardized according to Nomina Anatomica Veterinaria [16].

### 2.3. Cytogenetic Analysis

Chromosome preparations were obtained from an in vitro fibroblast culture. The cell culture was established from a skin sample collected postmortem. Cells were cultured using Dulbecco’s modified Eagle medium (DMEM), supplemented with 15% (*v*/*v*) fetal calf serum and 1% (*v*/*v*) penicillin/streptomycin at 37 °C in a humidified atmosphere of 5% CO_2_. To obtain the chromosome preparation, the cells were treated with 0.1 μg/mL Colcemid solution for 2 h. A standard cell harvesting procedure was applied using the hypotonic treatment and fixation step. The chromosomes were examined at an early passage (passages 1–2) using Giemsa staining, which facilitates the identification of a diploid chromosome number and recognition of the sex chromosomes. For each animal, at least thirty metaphase spreads were analyzed. Chromosomes were identified based on the one-arm morphology of the autosomes and the biarmed morphology of the sex chromosomes. The slides were examined with an epifluorescence Nikon E600 Eclipse microscope (Nikon, Tokyo, Japan) equipped with a cooled charge-coupled device CCD digital camera and Lucia software v.1.

### 2.4. Molecular Genetic Analysis

Genomic DNA was isolated from the skin tissue collected postmortem with the use of the commercial kit (Genomic Mini, A&A Biotechnology, Gdansk, Poland). Primers for the study genes were designed using Primer3Plus (Table S1). In terms of *ADAMTS20*, the PCR product (400 bp) overlaps the known 2 bp deletion (c.1360_1361delAA, p.Lys453Ilefs*3) described by Wolf et al. [3]. In the *DLX6* gene [15], the presence or absence of LINE-1 element insertion within intron 2 was verified using primers designed on the borderline of the insertion. When the insertion was not present, the 172 bp PCR product was expected, while no amplification was expected when the insertion was present (in a homozygote status). Additionally, the success of amplification was verified using the primers for the control fragment of the length of 222 bp (outside the insertion site) in the same PCR reaction (PCR-duplex). Based on the in silico comparison of cattle and canine *MYH3* gene sequences, a fragment (286 bp) of exon 21 was examined for each case. This fragment corresponded to the homologous region in the cattle genome where two CP-associated mutations—a 11-bp deletion and a single nucleotide A > G substitution—were found. The PCR amplifications were conducted under standard conditions following 2% agarose gel electrophoresis. To verify that there were no other nucleotide changes, the amplicons for *DLX6* (172 bp) were extracted from agarose gel using a GeneJET Gel Extraction Kit (Thermofisher Scientific, Waltham, MA, USA) following the manufacturer’s protocol. For the *ADAMTS20* and *MYH3*, the amplicons were initially purified with thermosensitive alkaline phosphatase and exonuclease I (ThermoFisher Scientific, Waltham, MA, USA). The subsequent steps were common to all the genes. They included amplification using BigDye Terminator v3.1 Cycle Sequencing (ThermoFisher Scientific, Waltham, MA, USA) and capillary electrophoresis on a 3130 Genetic Analyzer (Applied Biosystems, Waltham, MA, USA). Chromatograms were analyzed using SeqMan software v.1 (DNASTAR, Madison, WI, USA).

## 3. Results

**Figure 2:**
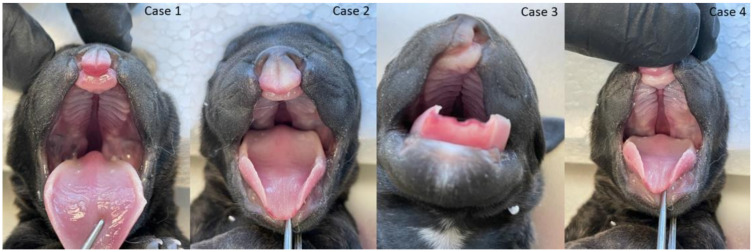
Cleft lip and palate and lip (CLP) in three puppies (three cases from the left side) and cleft lip (CL) in the fourth puppy (right).

The gross phenotypic evaluation revealed orofacial malformations in four puppies. Three of them had cleft lip and palate (CLP; Figure 2), and one had lip cleft (CL) only (Figure 2 and Figure S1). The dogs’ skull type was classified as mesocephalic. The primary and secondary palate cleft was found in each oral cavity. There were nine palatine rugae on the mucous membrane of each part of the disunited palate (Figure 2). The vomer was visible between the parts of the palate and showed no malformations in shape or length.

The arterial pattern for the region was typical for the species (Figures S2 and S3). The blood vessels supplying the area were the descending palatal artery (*arteria palatina descendens*) and the minor palatal artery (*arteria palatina minor*). Both vessels branched off from the maxillary artery (*arteria maxillaris*). The minor palatal artery supplied the soft palate. The descending palatine artery was a short trunk divided into the major palatine artery (*arteria palatina major*) and the sphenopalatine artery (*arteria sphenopalatina*). The major palatine artery supplied the hard palate, running halfway across the width of the palate from the major palatine foramen heading to the incisor portion of the palate. After penetrating the nasal cavity, the sphenopalatine artery is divided into the caudal, lateral, and septal nasal arteries (*arteriae nasales caudales*, *laterales et septales*).

**Figure 3:**
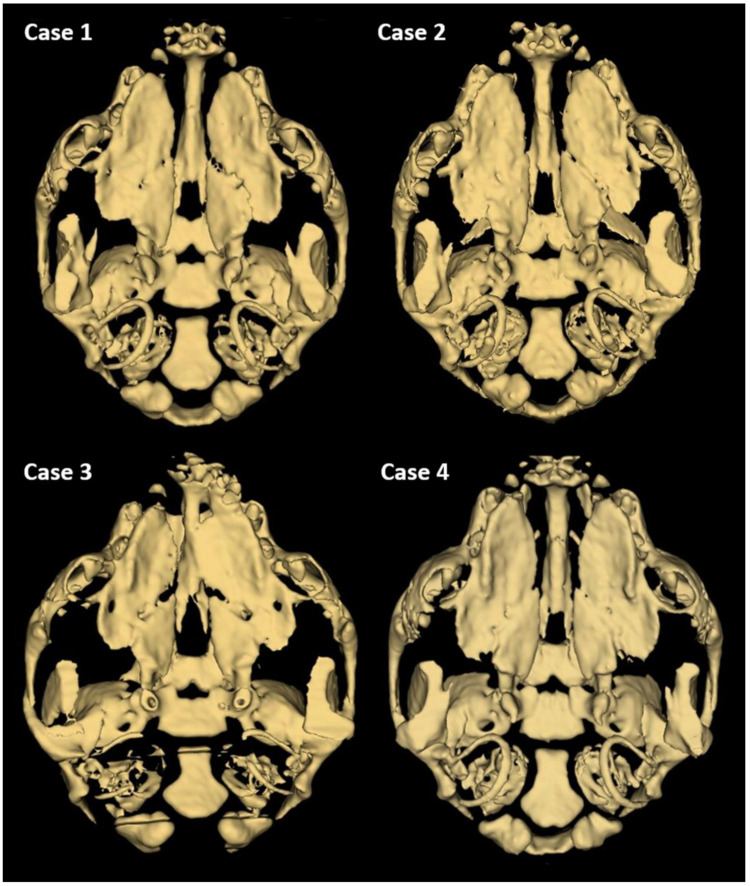
3D reconstruction of CT scan of the head of a one-day-old Staffordshire Bull Terrier dog with cleft palate. Individual differences in the unification of palatal processes of the hard palate are visible.

Further phenotyping was performed by CT imaging. CBCT scans provided a complete image of the hard palate (Figure 3). A detailed description of the palatal and lip abnormalities is included below. There was no malformation in the nasal septum, conchae, wall of the nasal cavity, tympanic bullae, or other anatomical structures of the head. Cases 1, 2, and 4 had complete CLP on both sides. On the left side, the horizontal plate of the palatine bone was fused with the palatal process of the maxilla. On the right side, those structures were fused completely only in case 4. The incisive bone was bilaterally reduced with no palatal process. Case 3 had a complete CP on the left side. On both sides, the horizontal plate of the palatine bone was fused with the palatal process of the maxilla. On the right side, the horizontal plate of the maxilla is normally fused with the palatal process of the incisive bone with a properly developed palatine fissure. This part of the hard palate was fused with the vomer correctly. On the left side, the incisive bone was reduced.

Since congenital malformations can be caused by abnormal chromosome complements, cytogenetic evaluation of the affected puppies was performed. An analysis of Giemsa-stained chromosome preparations revealed a normal chromosome set of 78,XY in three puppies and 78,XX in one puppy (Figure S4). Chromosomal sex was consistent with phenotypic sex. No chromosome aneuploidy or gross chromosomal abnormalities were found.

Molecular analysis of three candidate genes for CLP employed PCR amplification, electrophoresis, and DNA Sanger sequencing. The analysis focused on detecting known DNA variants associated with CLP. In *ADAMTS20*, an expected causative 2 bp deletion was not found in the four puppies (Figure S5). PCR analysis of *DLX6* showed the presence of a 172 bp fragment—evidence of a lack of LINE-1 insertion in the homozygous form (Figure S6); sequencing did not reveal additional nucleotide changes. Sequence analysis of *MYH3* did not reveal the presence of potential variants (11-bp deletion and A > G substitution) in any of the puppies (Figure S7).

## 4. Discussion

Although cleft palate is one of dogs’ most common congenital malformations, detailed morphological evaluation of the head by computed tomography has been rare and limited to selected breeds [17]. A CP can range from a simple fissure involving only a small portion of the caudal soft palate to complex defects involving the soft palate, the hard palate caudal to the palatine fissures, and the vomer in the nasal cavity [18,19]. CP has been recognized as a factor that can lead to death within the first three days of life of puppies belonging to large dog breeds. Typical symptoms of CP include difficulties in food intake, choking, sneezing, coughing, and nasal discharge [5]. Since CP can be treated surgically [20], it is essential to assess a detailed morphology of the cleft before surgery to use the best technique and to fully evaluate the spectrum of possible postoperative complications [21].

In the present study, we described a unique finding of four full-sibs with CLP or CP in a single litter of seven puppies of Staffordshire Bull Terriers. CLP has been previously described in this breed, but detailed information on the morphology of the malformations has been scarce [10]. The puppies examined in this study showed diversity regarding the morphological changes in lips and palate. Pankowski et al. [17] also reported phenotypic variation of palatal defects in ten one-day-old puppies representing six breeds (four English Bulldogs, a French Mastiff, a Golden Retriever, a Basset Hound, a Yorkshire Terrier, a German Shepherd, and a West Highland White Terrier). Five cases of CP, three cases of bilateral CLP, and one case each of unilateral CLP and unilateral CL were observed among the ten affected puppies. In the present study, we did not observe any deformations other than the ones described above.

Following the classification of orofacial abnormalities described by Moura et al. [10], the puppies reported in this study fell into group III (cleft covering primary and secondary palate: total or partial impairment). Clefts involving just the lip without the palate may not require surgical intervention since they have only aesthetic effects. Clefts involving deeper structures like the hard or soft palate may be considered for surgical reparation. Surgery can be performed in puppies with fully grown incisors and canine teeth; dogs that cannot be fed with assistance thus do not qualify for treatment [22]. The arterial vascularization of the hard palate in the four puppies examined here did not differ from the vascular system described in healthy dogs. The same arrangement of vessels and area of supply is shown in the available literature [23,24].

CT imaging can improve CP diagnostic protocols by providing images of bone tissue in addition to those of the soft tissue of the oral cavity. None of the craniomaxillofacial anomalies found in previous studies (i.e., hypoplastic tympanic bullae, hypoplastic nasal turbinates, malformed vomer, or nadal septum) were present in a recent study [25]. CT examination is an important part of surgical planning. Thus, detailed anatomical characteristics of the CL and palate in Staffordshire Bull Terrier dogs may be helpful for surgical reconstruction of this anomaly in dogs. CT scans can help identify the incorrectly developed structures and determine the surgical technique. Several techniques are used to repair CLP. Some employ musocal or mucoperiosteal flaps to close the clefts, while more advanced techniques involve using bone grafts and prostheses to treat larger clefts [10]. Other techniques involve the use of mesenchymal stem cells of the iliac bone with hydroxyapatite particles [26].

It is known that polygenic nonsyndromic forms of orofacial clefts in humans can be associated with predisposing germinal DNA variants or fetal exposure to teratogenic factors, while syndromic forms can be caused by chromosomal aberrations or single gene mutation [27]. Chromosome analysis is commonly used in studies of congenital malformation. All the puppies examined here, although they had nonsyndromic clefts, were subjected to cytogenetic analysis: normal chromosome sets (78,XX or 78,XY) were observed, allowing us to rule out aneuploidy as a cause of the abnormality.

In our molecular study, we selected three candidate genes in which causative mutations for CLP were found. Mutations in *DLX6* and *ADAMTS20* were identified in affected Nova Scotia duck-tolling retrievers [3,15]. It should be mentioned that some of the affected NSDTR dogs also had other anomalies, such as syndactyly (webbed or conjoined digits) in the paws or shortened mandibles; these may indicate that the clefting was syndromic. The *ADAMTS20* mutation was classified as autosomal recessive with variable expressivity [3]. LINE-1 insertion in the *DLX6* gene did not explain a series of NSDTR cases with CL. It has been shown that both causative mutations segregated independently within the NSDTR dog breed, though in two NSDTR cases, the causative DNA variant was not identified [3]. This indicates the genetic heterogeneity of the orofacial cleft within this breed. Our study did not reveal the presence of any DNA variants of either gene in the puppies; this agrees with the findings of a previous study [3] that these variants are specific to the NSDTR breed. However, it should be pointed out that our analysis only facilitated the detection of the homozygous genotype for LINE-1 insertion in *DLX6*. We additionally examined the *MYH3* gene, in which causal mutations have been observed in Limousine cattle (1-bp nonsynonymous substitution and a 11-bp frameshift deletion), as reported by Vaiman et al. [6]. Mutations of this gene are responsible for Freeman–Sheldon syndrome in humans [28], mainly manifested by microstomia (undersized mouth) and pursed lips. Our analysis did not identify the DNA variants in the *MYH3* gene fragment reported by Vaiman et al. [6].

## 5. Conclusions

The occurrence of craniofacial abnormalities in over 50% of puppies with a normal set of chromosomes (78,XX or 78,XY) from the same litter suggests that exposure to harmful environmental factors during pregnancy may be the most probable cause of the malformation [29,30]. However, other factors—including variants of other genes and epigenetic mechanisms [31]—should also be considered.

## Data Availability

The data presented in this study are available on request from the corresponding author.

## Supplementary Materials

The supporting information can be downloaded at https://www.mdpi.com/article/10.3390/ani13172749/s1, Table S1. PCR primers were used to study the *ADAMTS20*, *DLX6* and *MYH3* genes. Figure S1. Bilateral cleft of the lip in Staffordshire Bull Terrier puppy (case 4). Black arrows indicate the clefts. Figure S2. Anatomical preparation of the head of the Staffordshire Bull Terrier dog (case 4). The arterial vessels are visible due to the latex injection technique. 1: cleft of the lip; 2: septal nasal artery; 3: vomer; *: disunited parts of the palate. Figure S3. Latex preparation of the palatal region of the Staffordshire Bull Terrier puppy (case 1). 1: maxillary artery; 2: descending palatine artery; 3: infraorbital artery; 4: major palatine artery; 5: septal nasal artery. Figure S4. Representative metaphase spreads showing normal chromosome complement for male and female dogs in the puppies. Sex chromosomes are indicated by arrows (X: red, Y: blue). Figure S5. Sequencing of the candidate fragment of the *ADAMTS20* gene in the dogs, showing the lack of 2 bp deletion (in the frame). Figure S6. Agarose gel electrophoresis for the fragments in the *DLX6* gene after PCR-duplex (222 bp: control amplicon; 172 bp fragment amplified if the LINE-1 insertion is not present in a homozygote status). L: DNA Ladder; 1–4: the affected puppies; 0: negative control (no DNA template). Figure S7. Sequencing of the candidate region of the *MYH3* gene in the four dogs showed the lack of 11-bp deletion and A > G substitution (in frames).

## References

[1] Roman N., Carney P.C., Fiani N., Peralta S. (2019). Incidence Patterns of Orofacial Clefts in Purebred Dogs. PLoS ONE.

[2] Vieira A.R., Avila J.R., Daack-Hirsch S., Dragan E., Félix T.M., Rahimov F., Harrington J., Schultz R.R., Watanabe Y., Johnson M. (2005). Medical Sequencing of Candidate Genes for Nonsyndromic Cleft Lip and Palate. PLoS Genet..

[3] Wolf Z.T., Brand H.A., Shaffer J.R., Leslie E.J., Arzi B., Willet C.E., Cox T.C., McHenry T., Narayan N., Feingold E. (2015). Genome-Wide Association Studies in Dogs and Humans Identify ADAMTS20 as a Risk Variant for Cleft Lip and Palate. PLoS Genet..

[4] Mulvihill J.J., Mulvihill C.G., Priester W.A. (1980). Cleft Palate in Domestic Animals: Epidemiologic Features. Teratology.

[5] Shaw D.H., Ihle S.L. (2013). Small Animal Internal Medicine.

[6] Vaiman A., Fritz S., Beauvallet C., Boussaha M., Grohs C., Daniel-Carlier N., Relun A., Boichard D., Vilotte J.-L., Duchesne A. (2022). Mutation of the MYH3 Gene Causes Recessive Cleft Palate in Limousine Cattle. Genet. Sel. Evol..

[7] Shupe J.L., James L.F., Binns W., Keeler R.F. (1968). Cleft Palate in Cattle. Cleft Palate J..

[8] Lobodzinska A., Gruszczynska J., Max A., Bartyzel B.J., Mikula M., Mikula I., Grzegrzolka B. (2014). Cleft Palate in the Domestic Dog, Canis Lupus Familiaris–Etiology, Pathophysiology, Diagnosis, Prevention, and Treatment. Acta Sci. Polon. Zootech..

[9] Estevam M.V., Beretta S., Smargiassi N.F., Apparício M., Toniollo G.H., Pereira G.T. (2022). Congenital Malformations in Brachycephalic Dogs: A Retrospective Study. Front. Vet. Sci..

[10] Moura E., Pimpão C.T., Almasri M. (2017). Cleft Lip and Palate in the Dog: Medical and Genetic Aspects. Designing Strategies for Cleft Lip and Palate Care.

[11] Kelly K.M., Bardach J. (2012). Biologic Basis of Cleft Palate and Palatal Surgery. Oral and Maxillofacial Surgery in Dogs and Cats.

[12] Martinelli M., Palmieri A., Carinci F., Scapoli L. (2020). Non-Syndromic Cleft Palate: An Overview on Human Genetic and Environmental Risk Factors. Front. Cell Dev. Biol..

[13] Ludwig K., Böhmer A.C., Bowes J., Nikolić M., Ishorst N., Wyatt N., Hammond N., Gölz L., Thieme F., Barth S. (2017). Imputation of orofacial clefting data identifies novel risk loci and sheds light on the genetic background of cleft lip ± cleft palate and cleft palate only. Hum. Mol. Genet..

[14] Awotoye W., Mossey P.A., Hetmanski J.B., Gowans L.J.J., Eshete M.A., Adeyemo W.L., Alade A., Zeng E., Adamson O., Naicker T. (2022). Whole-Genome Sequencing Reveals De-Novo Mutations Associated with Nonsyndromic Cleft Lip/Palate. Sci. Rep..

[15] Wolf Z.T., Leslie E.J., Arzi B., Jayashankar K., Karmi N., Jia Z., Rowland D.J., Young A., Safra N., Sliskovic S. (2014). A LINE-1 Insertion in DLX6 Is Responsible for Cleft Palate and Mandibular Abnormalities in a Canine Model of Pierre Robin Sequence. PLoS Genet..

[16] International Committee on Veterinary Gross Anatomical Nomenclature (2017). Nomina Anatomica Veterinaria.

[17] Pankowski F., Paśko S., Max A., Szal B., Dzierzęcka M., Gruszczyńska J., Szaro P., Gołębiowski M., Bartyzel B.J. (2018). Computed Tomographic Evaluation of Cleft Palate in One-Day-Old Puppies. BMC Vet. Res..

[18] Xiao Y., Jiao S., He M., Lin D., Zuo H., Han J., Sun Y., Cao G., Chen Z., Liu H. (2022). Chromatin Conformation of Human Oral Epithelium Can Identify Orofacial Cleft Missing Functional Variants. Int. J. Oral Sci..

[19] Lan Y., Jiang R. (2022). Chapter Two—Mouse Models in Palate Development and Orofacial Cleft Research: Understanding the Crucial Role and Regulation of Epithelial Integrity in Facial and Palate Morphogenesis. Curr. Top. Dev. Biol..

[20] Conze T., Ritz I., Hospes R., Wehrend A. (2018). Management of Cleft Palate in Puppies Using A Temporary Prosthesis: A Report of Three Cases. Vet. Sci..

[21] Rossell-Perry P., Caceres Nano E., Gavino-Gutierrez A.M. (2014). Association Between Palatal Index and Cleft Palate Repair Outcomes in Patients with Complete Unilateral Cleft Lip and Palate. JAMA Fac. Plast. Surg..

[22] Fiani N., Verstraete F.J.M., Arzi B. (2016). Reconstruction of Congenital Nose, Cleft Primary Palate, and Lip Disorders. Vet. Clin. Small Anim. Pract..

[23] Nickel R., Schwarz R. (1963). Vergleichende Betrachtung Der Kopfarterien Der Haus-Säugetiere (Katze, Hund, Schwein, Rind, Schaf, Ziege, Pferd). Zentralbl. Veterinärmed. Reihe A.

[24] Carroll K.A., Mathews K.G. (2020). Ligation of the Maxillary Artery Prior to Caudal Maxillectomy in the Dog—A Description of the Technique, Retrospective Evaluation of Blood Loss, and Cadaveric Evaluation of Maxillary Artery Anatomy. Front. Vet. Sci..

[25] Nemec A., Daniaux L., Johnson E., Peralta S., Verstraete F.J.M. (2015). Craniomaxillofacial Abnormalities in Dogs with Congenital Palatal Defects: Computed Tomographic Findings. Vet. Surg..

[26] Kawano H., Kimura-Kuroda J., Komuta Y., Yoshioka N., Li H.P., Kawamura K., Li Y., Raisman G. (2012). Role of the Lesion Scar in the Response to Damage and Repair of the Central Nervous System. Cell Tissue Res..

[27] Askarian S., Gholami M., Khalili-Tanha G., Tehrani N.C., Joudi M., Khazaei M., Ferns G.A., Hassanian S.M., Avan A., Joodi M. (2023). The genetic factors contributing to the risk of cleft lip-cleft palate and their clinical utility. Oral Maxillofac. Surg..

[28] Beck A.E., McMillin M.J., Gildersleeve H.I.S., Shively K.M.B., Tang A., Bamshad M.J. (2014). Genotype-Phenotype Relationships in Freeman–Sheldon Syndrome. Am. J. Med. Genet. Part A.

[29] Molina-Solana R., Yáñez-Vico R.M., Iglesias-Linares A., Mendoza-Mendoza A., Solano-Reina E. (2013). Current concepts on the effect of environmental factors on cleft lip and palate. Int. J. Oral Maxillofac. Surg..

[30] Leśków A., Nawrocka M., Łątkowska M., Tarnowska M., Galas N., Matejuk A., Całkosiński I. (2019). Can contamination of the environment by dioxins cause craniofacial defects?. Hum. Exp. Toxicol..

[31] Butler M.G. (2002). Imprinting disorders: Non-Mendelian mechanisms affecting growth. J. Pediatr. Endocrinol. Metab..

