# Supplementary material for: Cleft Lip and Palate in Four Full-Sib Puppies from a Single Litter of Staffordshire Bull Terrier Dogs: An Anatomical and Genetic Study
Source: Animals (Basel). 2023 Aug 29;13(17):2749. doi: 10.3390/ani13172749 (PMC10486559; doi:10.3390/ani13172749)
Supplement: Supplementary file 1 [file animals-13-02749-s001.zip › animals-2532976-supplementary.pdf]

Supplementary Materials

Cleft lip and palate in four full-sib puppies from a single litter of Staffordshire Bull Terrier dogs: An anatomical and genetic study

Jakub J. Ruszkowski [1,†], Joanna Nowacka-Woszuk [2,†], Tomasz Nowak [2], Jedrzej Rozynek [2], Katarzyna Serwanska-Leja [1], Maciej Gogulski [3,4], Pawel Kolodziejski [5], Marek Switonski [2], Maciej Zdun [1,*], Izabela Szczerbal [2,*]

Table S1. PCR primers used in the study of the *ADAMTS20*, *DLX6* and *MYH3* genes.

Gene	Analyzed variant	Primers sequence (genomic location of the studied regions according to CanFam3.1)	Annealing temperature	Amplicon length
ADAMTS20	2 bp deletion (c.1360_1361delAA)	F: 5’ TGTTCCACATGATGATAGTTTTAAATG<br>R: 5’ CCCGGGTCTCTAGAATCACA<br>(chr27:10553360 - 10553759)	56 ºC	400 bp
DLX6	LINE 1 insertion in intron 2	F: 5’ ACCATCGCTTTCAGCAAACT<br>R: 5’ CTAGGCCCAGAATTCCTCCT<br>(chr14:22067918 - 22068089)	60 ºC	172 bp
	control amplicon	F: 5’ AAAACGGGGAAATCAGGTTC<br>R: 5’ ATCTGCAGGCAGGGTCAG<br>(chr14:22067831 - 22068052)	60 ºC	222 bp
MYH3	11-bp deletion and A>G substitution in exon 21	F: 5’ TGAAAACTTGTTGGATGCAGA<br>R: 5’ TGATTCACAATGAGCACACG<br>(chr5:34924947 - 34925232)	61 ºC	286 bp

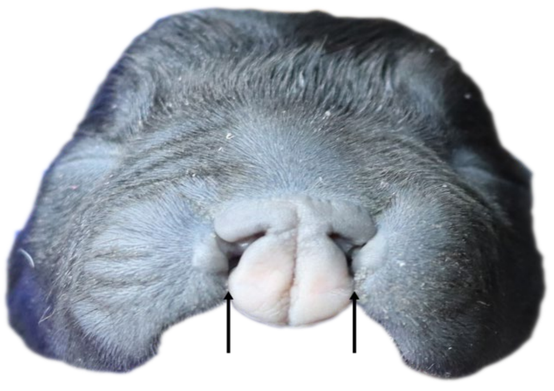

Figure S1. Bilateral cleft of the lip in Staffordshire Bull Terrier puppy (case 4). Black arrows indicate the clefts.

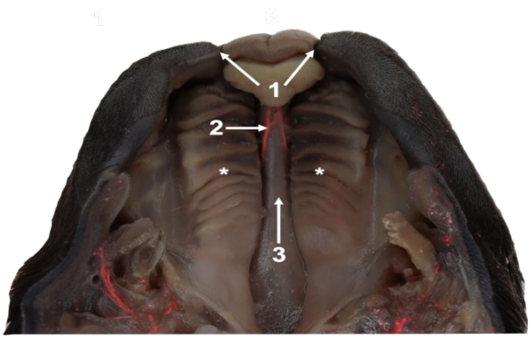

Figure S2. Anatomical preparation of the head of the Staffordshire Bull Terrier dog (case 4). The arterial vessels are visible due to the latex injection technique. 1: cleft of the lip; 2: septal nasal artery; 3: vomer; *: disunited parts of the palate.

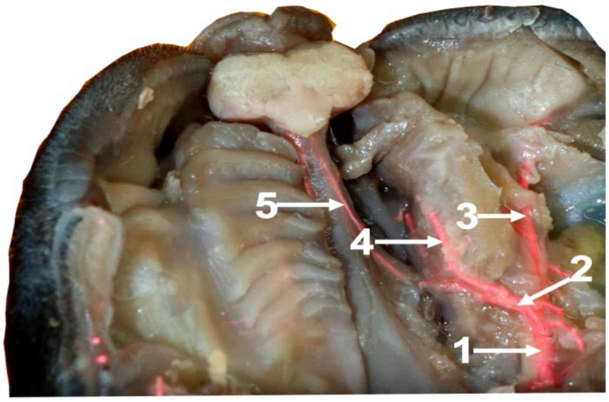

Figure S3. Latex preparation of the palatal region of the Staffordshire Bull Terrier puppy (case 1). 1: maxil-lary artery; 2: descending palatine artery; 3: infraorbital artery; 4: major palatine artery; 5: septal nasal artery.

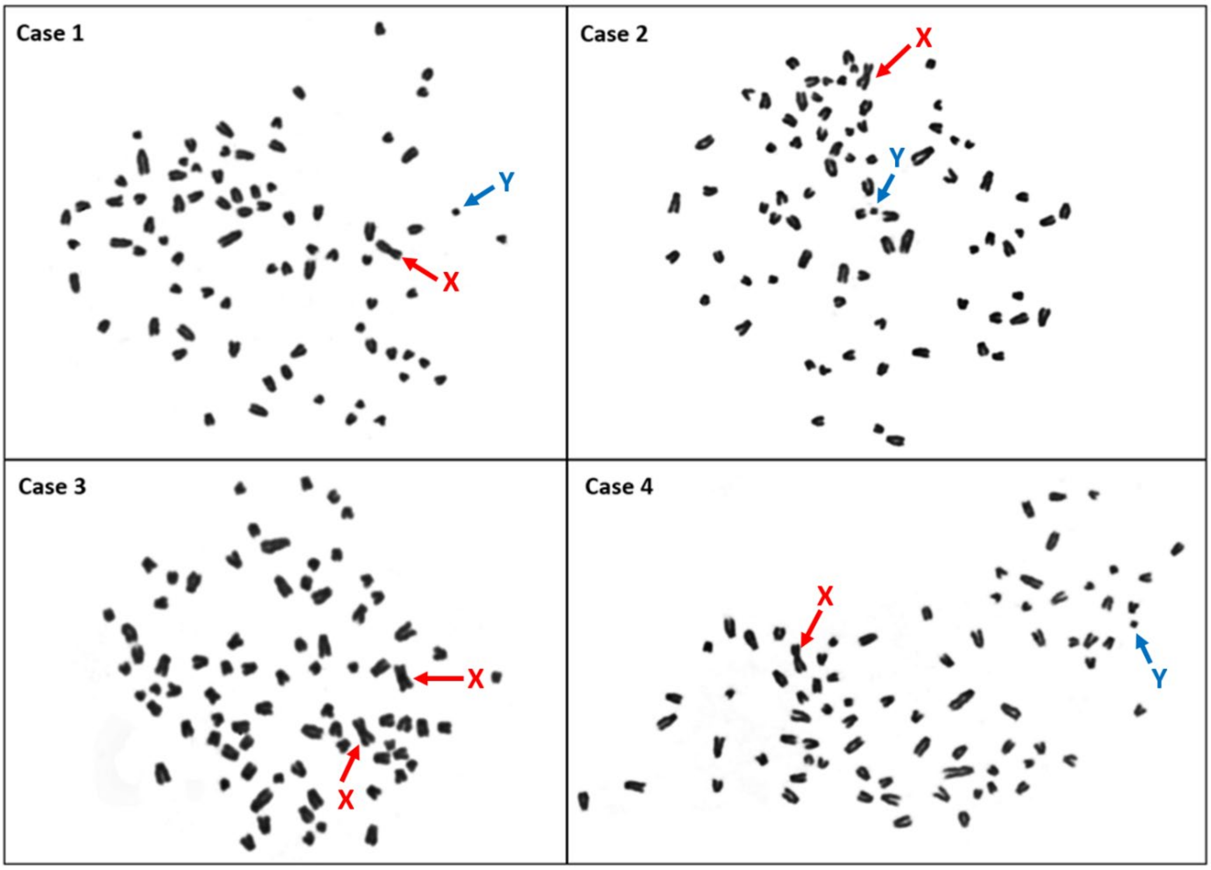

Figure S4. Representative metaphase spreads showing normal chromosome complement for male and fe-male dogs in the puppies. Sex chromosomes are indicated by arrows (X: red, Y: blue).

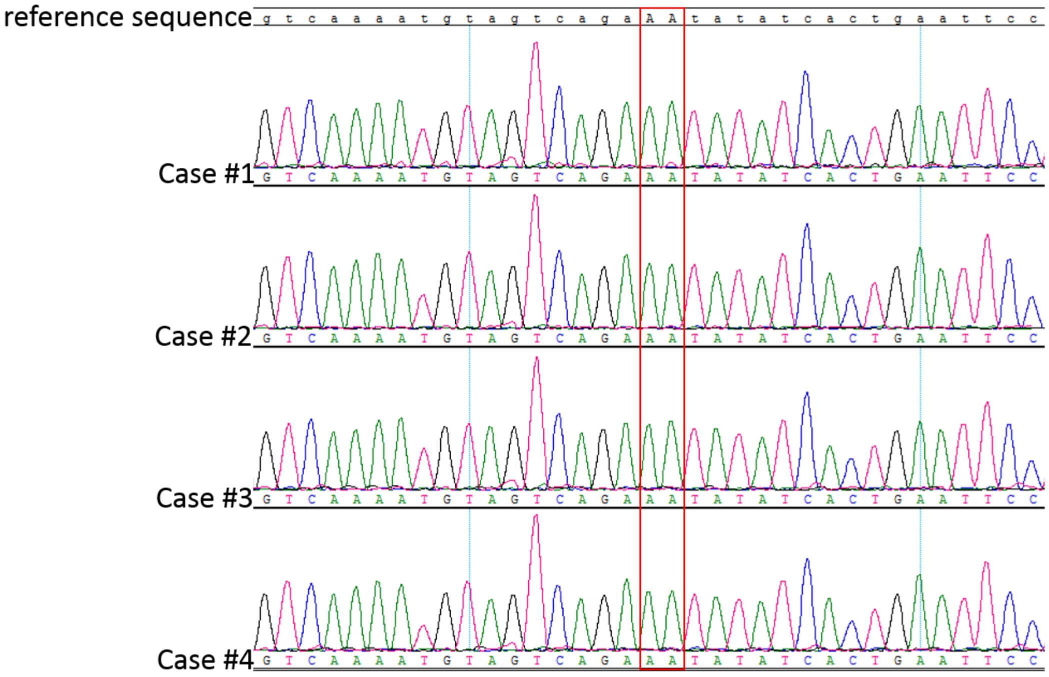

Figure S5. Sequencing of the candidate fragment of the *ADAMTS20* gene in the dogs, showing the lack of 2 bp deletion (in the frame).

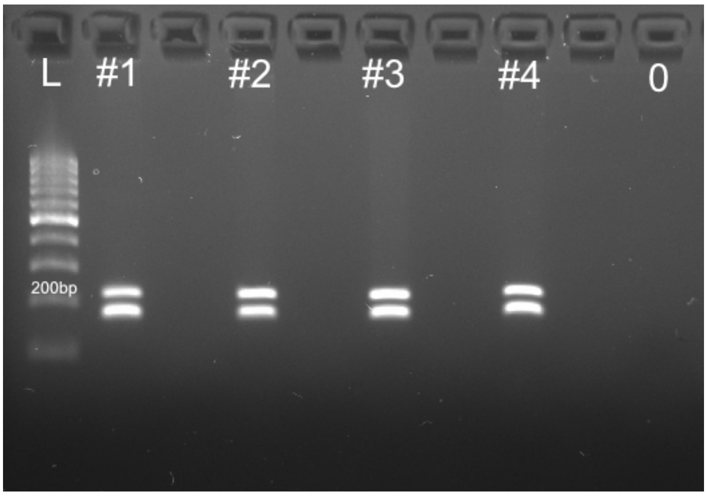

Figure S6. Agarose gel electrophoresis for the fragments in *DLX6* gene after PCR-duplex (222 bp: control amplicon; 172 bp fragment amplified if the LINE-1 insertion is not present in a homozygote sta-tus). L: DNA Ladder; 1–4: the affected puppies; 0: negative control (no DNA template).

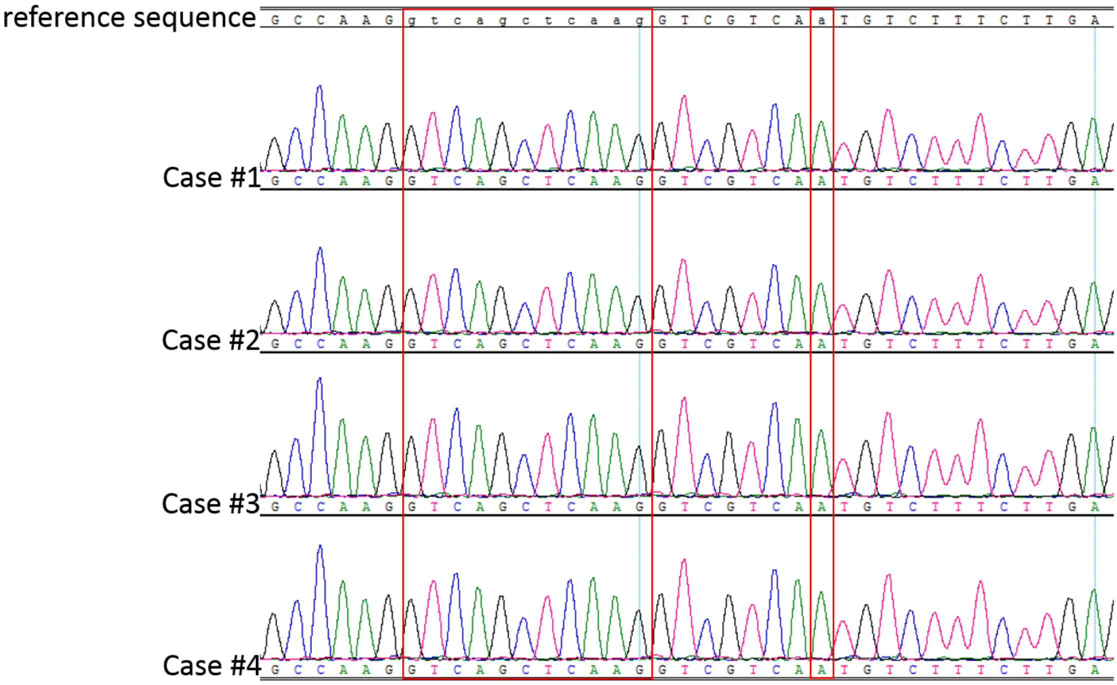

Figure S7. Sequencing of the candidate region of the *MYH3* gene in the four dogs showing the lack of 11-bp deletion and A>G substitution (in frames).
